# Home-Based Multimodal Exergame Prototype for Patients With Chronic Stroke: Cross-Sectional Content Validation and Prospective Single-Arm Feasibility Study

**DOI:** 10.2196/86873

**Published:** 2026-07-14

**Authors:** Puntarik Keawtep, Cattaleeya Sittichoke, Phattharakon Nitsaphawanit, Pattaratida Euanjit, Somporn Sungkarat

**Affiliations:** 1 Integrated Neuro-Musculoskeletal, Chronic Disease, and Aging Research Engagement Center (ICARE Center), Department of Physical Therapy, Faculty of Associated Medical Sciences, Chiang Mai University, 110 Intawaroros Rd, Sripoom, Chiang Mai, 50200, Thailand

**Keywords:** cardiovascular disease, exergame, stroke, health, rehabilitation

## Abstract

**Background:**

Patients with chronic stroke commonly experience motor and cognitive impairments, with diminished quality of life. Their participation in rehabilitation is often limited by long travel distances and low motivation. Given the health benefits of multimodal exercise, with the accessibility and engagement offered by interactive exergames, delivering such interventions in a remote format may help overcome common barriers, and enhance health outcomes.

**Objective:**

This study aimed to assess the content validity and feasibility of a prototype home-based multimodal exergame for patients with chronic stroke.

**Methods:**

This study comprised a cross-sectional study for content validity and preliminary usability evaluation (part 1), and a prospective single-arm feasibility study with blinded assessors (part 2). In part 1, the content validity of a prototype home-based multimodal exergame was evaluated by 5 experts. Moreover, 5 patients with chronic stroke were enrolled to evaluate the preliminary usage of the game prototype. Enjoyment, rating of perceived exertion, user feedback, satisfaction, and safety were assessed. In part 2, 9 patients with chronic stroke were enrolled to evaluate the feasibility of home-based multimodal exergame (mean age 53.67, SD 11.70 years, and mean time post stroke onset 75.00, SD 101.99 months). Participants performed the intervention for 60 min/day, 3 days/week, for 6 weeks. Feasibility outcomes, including adherence, adverse events, motor function, cognitive function, and enjoyment of the program, were determined.

**Results:**

In part 1, the content validity index for the overall program was 0.82. The average total enjoyment score was 47.80 out of 56 (SD 7.50), the rating of perceived exertion was 13.40 out of 20 (SD 0.55), the satisfaction score was 8.60 out of 10 (SD 1.34), and no adverse events were reported. In part 2, after the 6-week intervention, positive changes were observed in global cognition (mean difference=−3.44, 95% CI −5.66 to −1.23; *P*=.007) and executive function (mean difference=38.43, 95% CI 3.78-73.08; *P*=.03) compared with baseline. Participants reported a consistently high level of exercise enjoyment throughout the training period. The average exercise adherence rate was 85.19%, and no adverse events were reported throughout the training.

**Conclusions:**

These findings suggest that the prototype home-based multimodal exergame was a valid, enjoyable, accessible, and user-friendly rehabilitation approach. Unlike conventional home exercise programs, this multimodal exergame integrates physical and cognitive training into an interactive remote rehabilitation platform for individuals with chronic stroke. The intervention showed potential benefits for cognitive outcomes, maintained enjoyment, and promoted exercise adherence, and reported no adverse events throughout the intervention period. These findings provide preliminary evidence that home-based multimodal exergame may support long-term rehabilitation participation. Importantly, the program may address barriers to stroke rehabilitation, including transportation limitations, restricted access to rehabilitation services, and low long-term adherence, particularly among individuals living in underserved or remote areas.

## Introduction

Globally, stroke is recognized as a leading cause of mortality and a major contributor to the burden on public health systems [1]. Findings from the Global Burden of Disease database revealed that disability-adjusted life years due to stroke increased by 32% from 1990 to 2019, reaching approximately 140 million [2]. Additionally, approximately 30%‐40% of survivors of stroke experience cognitive or motor impairments that contribute to long-term disability [3]. These poststroke impairments have been shown to adversely affect quality of life and functional independence, increase dependence in activities of daily living, and limit social participation [3,4]. Therefore, comprehensive rehabilitation strategies are essential to address multiple domains of recovery and improve health outcomes in survivors of stroke.

It has been well-established that poststroke rehabilitation and multimodal exercise are essential in improving health outcomes and reducing long-term complications [5,6]. Several studies have demonstrated that multimodal or multicomponent exercise programs incorporating balance, coordination, resistance, and aerobic training can enhance various aspects of physical function and activities of daily living [7-9]. Although the health benefits of physical activity and exercise are well-recognized, several survivors of stroke are physically inactive [10]. A prospective longitudinal cohort study reported that nearly 40% of participants were physically inactive at the 1-year follow-up after stroke [11]. Additionally, adherence to rehabilitation programs tends to decline over time, particularly in long-term interventions in survivors of stroke [12]. The limitation of engagement in physical activity and rehabilitation among patients with stroke is partly due to long travel distances, limited transportation access, shortages of health care providers, and low motivation [13,14]. These barriers can substantially limit long-term rehabilitation participation, underscoring the need for interventions that are accessible, engaging, and effective in promoting motivation and adherence. To address these challenges, particularly the need for long-term engagement, the integration of augmented feedback has emerged as a crucial strategy for improving both motor outcomes and motivation in individuals with cerebrovascular injury [15-17]. Clinical studies in patients with stroke have also indicated that training with augmented feedback improved walking speed and distance [18-20]. Therefore, multimodal exercise that is accessible, safe, enjoyable, and provides augmented feedback may promote health benefits and improve adherence to regular physical activity among patients with chronic stroke.

Technology-based interventions (eg, exergame) have been incorporated into medical treatments, offering accessible and motivating rehabilitation strategies [15,21-23]. Research evidence demonstrated that exergaming has positive impacts on balance and functional mobility in patients with chronic stroke [15]. Apart from the beneficial effects of exergaming on physical health, its interactive characteristics promote motivation among users, leading to greater adherence compared with regular exercise [21,22], and making it enjoyable and effective for patients with stroke [24]. Previous studies demonstrated that home-based exergame improved physical outcomes and health-related quality of life in older adults [5] and patients with stroke [25,26]. A recent study by our research group demonstrated that home-based interactive physical-cognitive exergame via a computer vision-based platform was usable and enjoyable for older adults both with and without cognitive impairment [23]. Despite these promising findings, evidence regarding home-based multimodal exergame interventions specifically designed for individuals with chronic stroke remains limited. Existing rehabilitation studies in survivors of stroke have largely focused on physical exercise interventions [9] and clinic-based rehabilitation programs [9,27]. Furthermore, recent evidence has highlighted the importance of developing strategies that promote long-term exercise persistence and adherence among survivors of stroke [28]. To the best of our knowledge, studies investigating multimodal exercise delivered via an exergame platform for patients with chronic stroke in home-based or remote settings remain limited. Therefore, this study aimed to evaluate the content validity and feasibility of a novel home-based multimodal exergame integrating physical-cognitive exercise, gamification, augmented feedback, and computer vision-based monitoring for individuals with chronic stroke. Given the well-established health benefits of multimodal exercise and the increasing accessibility of technology-based interventions, it is hypothesized that a home-based multimodal exergame would be valid and feasible for individuals with chronic stroke.

## Methods

### Study Design

This study consists of two parts: (1) the evaluation of the content validity index and the preliminary usage of a prototype home-based multimodal exergame using a cross-sectional study design, and (2) the evaluation of the feasibility of the home-based multimodal exergame for patients with chronic stroke using a prospective single-group study design.

### Inclusion and Exclusion Criteria

The inclusion criteria were (1) diagnosis of ischemic or hemorrhagic stroke at least 6 months before enrollment; (2) age between 20 and 80 years; (3) normal cognitive function, as determined by the Mental State Examination T10 score [29,30]; and (4) ability to see a notebook monitor screen from a distance of at least 60 cm, with or without corrective lenses. The exclusion criteria were (1) severe spasticity (Modified Ashworth Scale ≥3), (2) neglect or impaired communication, and (3) any comorbidity or complication that precluded participation in the training program.

### Participant Characteristics and Sampling Procedures

The study participants consisted of 2 groups involved in different phases of the study: expert reviewers in study part 1 and patients with stroke in study parts 1 and 2. The expert panel was recruited to evaluate the content validity of the prototype home-based multimodal exergame. Moreover, 5 experts with experience in game and web-application design, health innovation, stroke rehabilitation, neurocognition, and physical-cognitive training participated in the content validation process.

For the preliminary usability evaluation and feasibility study, demographic characteristics of the participants, including age, sex, time post onset, paretic side, and education level, were recorded. Patients with chronic stroke were recruited using convenience sampling through online and offline advertising to local communities, hospitals, rehabilitation centers, and social media advertisements. Participant screening and outcome assessments were conducted by blinded assessors.

### Sample Size, Power, and Precision

As this study was designed as a preliminary usability and feasibility study, a formal power analysis was not performed. The intended and achieved sample sizes were 5 participants with chronic stroke for the preliminary usability evaluation and 9 participants for the feasibility study. Sample sizes were determined pragmatically based on feasibility and the exploratory nature of the study. No interim analyses or stopping rules were applied.

### Procedure

**Figure 1.**
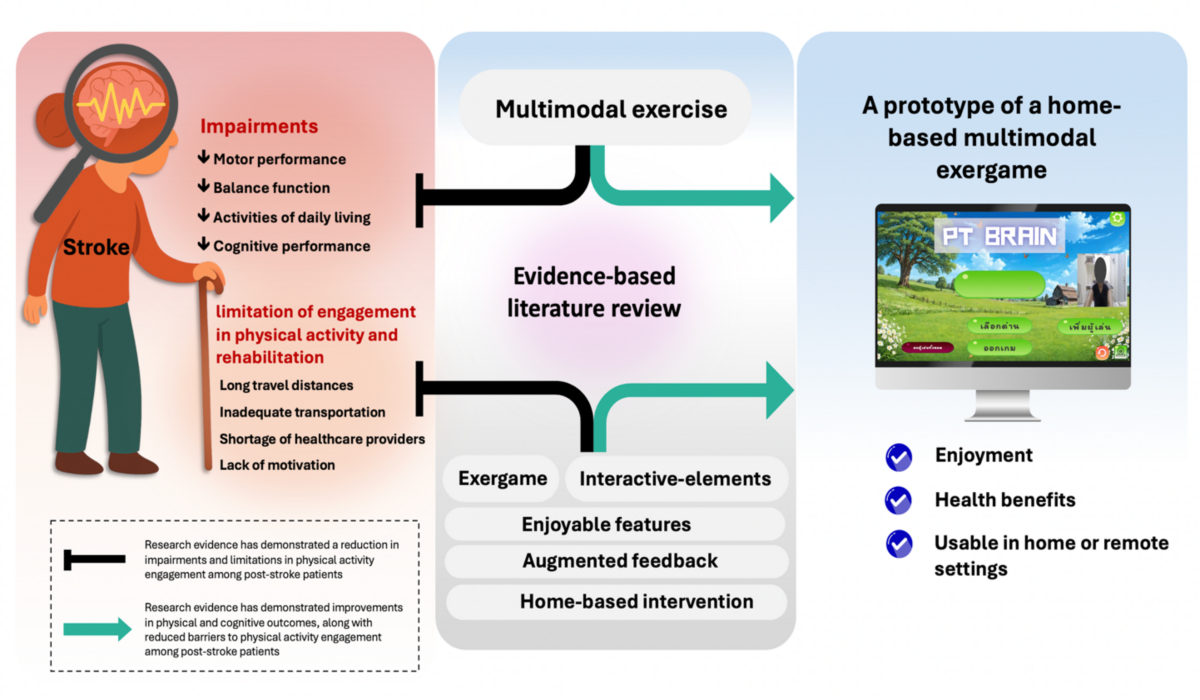
The summary of the design and characteristics of home-based multimodal exergame for patients with chronic stroke.

**Figure 2.**
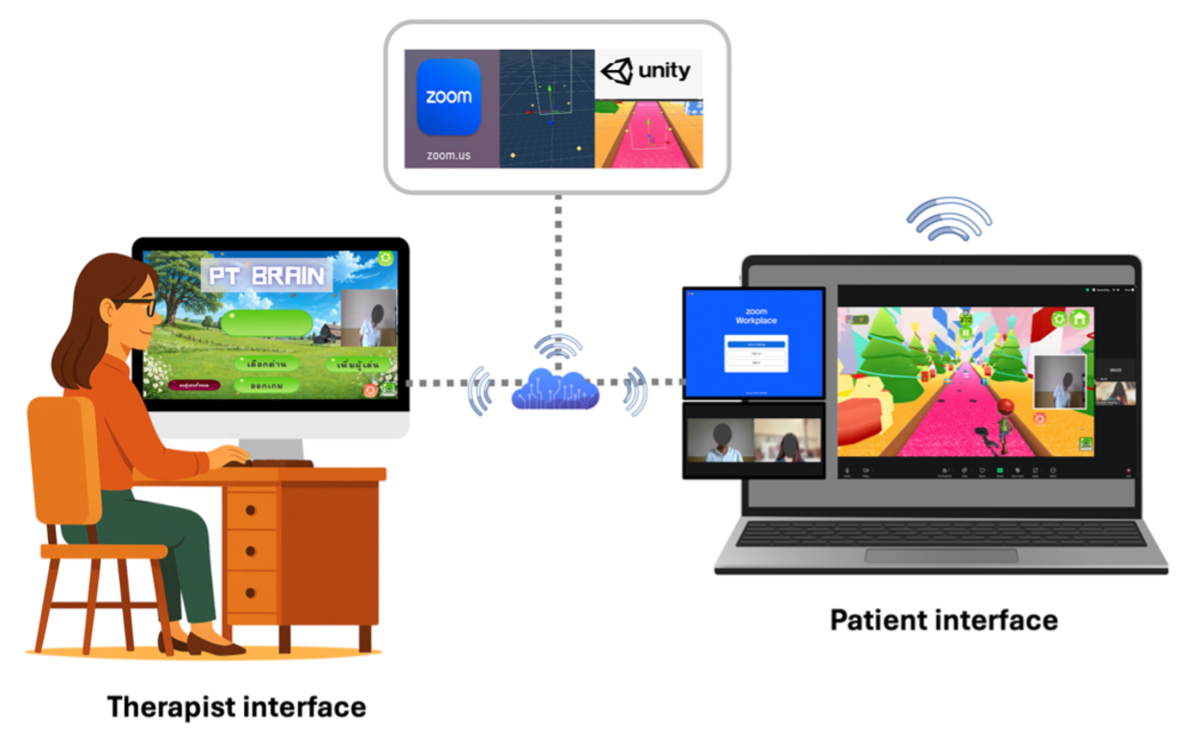
A framework of home-based multimodal exergame for patients with chronic stroke at home or in remote locations.

A novel home-based multimodal exergame was developed specifically for patients with chronic stroke by integrating the existing evidence from the literature [31,32] and adapted from our previous studies [33,34]. Additionally, a human-centered design methodology was applied to the development of the game-based exercise prototype [35], incorporating brainstorming sessions with the research team. Based on the synthesized evidence, a multimodal exergame-based intervention was applied to promote health performance, especially physical and cognitive aspects, among patients with stroke. The prototype of the multimodal exergame consists of physical exercises combined with cognitive training, including seated exercises, sit-to-stand training, proprioceptive neuromuscular facilitation (PNF) pattern-based exercises, balance training, aerobic exercise, and cognitive training targeting attention, memory, and executive function. Overall movements in multimodal exercise included shoulder flexion and extension, shoulder horizontal abduction and adduction, elbow flexion and extension, scapular protraction, lateral trunk flexion, hip flexion with knee flexion, hip abduction and adduction with knee flexion, knee flexion and extension, ankle dorsiflexion and plantarflexion. Gamification principles were integrated into the exergame to enhance user engagement, motivation, and adherence during exercise sessions. The overall structure and features of the game, including its objectives, gameplay mechanics, and graphical user interface, were systematically integrated and refined based on practical considerations to develop a functional prototype of the game-based training system. The exergame incorporated gamification features, including goal setting, rewards, interactive elements, and cognitive engagement, along with augmented feedback delivered through visual and auditory cues. Visual feedback (eg, on-screen movement tracking and progress bars) and auditory feedback (eg, sounds indicating success or errors) were integrated into the game-based design. Game-based concepts were applied to develop the digital exercise prototype using the Unity 3D game engine, incorporating computer vision techniques based on BlazePose for real-time pose estimation and user-system interaction. The system operates on a standard personal computer or notebook equipped with a webcam, without requiring additional wearable sensors. The system is semiautomated, enabling motion tracking and feedback without continuous therapist input. User-system interaction and feedback are achieved through real-time tracking of user movements, with feedback provided during gameplay as well as performance outcomes (eg, scores and errors) at the end of each game. The summary of the design and characteristics of home-based multimodal exergame is presented in Figure 1. Moreover, the novel home-based multimodal exergame includes a camera feature that displays participants’ movements, which can be monitored remotely through software such as Zoom (Zoom Communications) meetings. This functionality enables health care providers to remotely observe and assess the rehabilitation progress of patients with stroke at home or in remote locations, as illustrated in Figure 2.

The content validity of the developed intervention was assessed to determine whether its components accurately represent the intended construct for a specific clinical or research purpose. This evaluation was conducted by a panel of experts who rated each item based on its relevance and representativeness within the content domain. The content validity of the program was assessed by 5 experts who have expertise in the game and web-application design, innovation for health, stroke rehabilitation, neurocognition, and physical-cognitive training using the item-objective congruence (IOC) method. An overall IOC score greater than 0.75 for the intervention program was considered an acceptable content validity [36].

For the preliminary usability evaluation, written informed consent was obtained from all eligible participants before study participation. Demographic characteristics of participants, including age, sex, time post onset, paretic side, and education level, were recorded. A total of 5 patients with chronic stroke were enrolled to evaluate the preliminary usage of the game prototype. Participants meeting the eligibility criteria performed multimodal exergame. The multimodal exergame included a 10-minute warm-up and cool-down period, and 50 minutes of exercise. Participants were instructed to attend 2 sessions in a single visit to the research center. In session 1, participants were familiarized with the exergame via an in-game tutorial that provided detailed instructions on the correct performance of each exercise program. Following the tutorial, participants practiced a short sample of the exergame under the supervision of a physiotherapist. After the familiarization session, all participants were asked to rest until their perceived fatigue returned to baseline levels. In session 2, participants performed the multimodal exergame in the training room under the supervision of physiotherapists who remotely monitored them from the monitoring room. During exercise, participants were instructed to share their exergame desktop screen with the therapist via the Zoom meeting application. Additionally, the camera feature and computer vision-based system in the developed multimodal exergame allowed therapists to monitor exercise accuracy and safety.

After evaluating the content validity index and the preliminary usage of prototype home-based multimodal exergame, consensus from the expert and research team on the concept and design, along with comments and feedback, was used to refine the final version of the home-based multimodal exergame. The finalized version of the home-based multimodal exergame was used to evaluate the feasibility of the program in part 2 of the study.

For the evaluation of the feasibility of the home-based multimodal exergame, written informed consent was obtained from all eligible participants before participation in the study. This feasibility study was a prospective single-arm trial with blinded assessors conducted over a 6-week home-based intervention. A total of 9 patients with chronic stroke were recruited using convenience sampling to evaluate the feasibility of the exergame, applying the same inclusion and exclusion criteria as in part 1. Participants who met the eligibility criteria performed the intervention for 60 min/day, 3 days/week for 6 weeks. The home-based multimodal exergame is designed to be performed primarily in a seated position, with progression to functional tasks. The intervention comprises seated exercises, sit-to-stand training, PNF pattern-based exercises, balance training, aerobic exercise, and cognitive training. The multimodal exercise incorporates a range of joint movements and motor patterns involving the upper extremities, lower extremities, and trunk, with an emphasis on coordinated and functional movements such as alternating upper and lower limb movements, multidirectional reaching, weight shifting, trunk control, and sit-to-stand transitions. All exercises were performed for 5 repetitions per set for 2 sets, followed by 2 combined exercise sets (COMBO-SETs). The first COMBO-SET comprised alternating arm and leg lifts combined with forward arm and leg extensions, performed for 1 minute in level 1 and 2 minutes in level 2. The second COMBO-SET consisted of alternating boxing movements combined with alternating heel-toe tap steps, performed for 40 seconds in level 1 and 80 seconds in level 2. In addition, preparatory sit-to-stand training and sit-to-stand training were performed for 5 repetitions per set for 2 sets in level 1 and 3 sets in level 2. Cognitive tasks are embedded within the exergame to simultaneously engage attention, memory, and executive function. A summary of the home-based multimodal exergame is presented in Table 1. Before the home-based training, participants were asked to practice with a researcher to ensure they could perform the intervention correctly and safely. During the home-based intervention, participants were instructed to self-monitor their vital signs (eg, heart rate), rating of perceived exertion (RPE), and any adverse events. Participants were educated to recognize warning signs such as dizziness, loss of balance, or unusual symptoms, and were instructed to immediately stop exercising and notify the research team if such symptoms occurred. A researcher (PN) met with the participants via Zoom at the end of weeks 1, 2, and 4 to monitor their training. Each participant was assigned a unique Zoom ID, and all sessions were conducted on a one-to-one basis to ensure privacy and confidentiality. During the monitoring sessions, participants were instructed to share their screens with the researcher. In addition, the integrated camera function and computer vision-based system enabled real-time monitoring of exercise performance, allowing the researcher to assess movement accuracy and safety. The training sessions were recorded with participants’ consent. In addition, all recorded and collected data were anonymized and securely stored. Family members were permitted to stand beside participants during exercises at home.

**Table 1.** A summary of the consensus features of exergame prototype (characteristics and descriptions of the exergame prototype) for patients with chronic stroke.

Characteristics	Descriptions
Overall features	
Program topic	Prototype of a home-based multimodal exergame
Targeted users	Patients with chronic stroke
Core components	A multimodal exergame designed for home-based or remote use, consisting of (1) warm-up and cool-down phases (10 min), (2) main exercise phase (50 min) including seated exercises, sit-to-stand training, PNF^a^ pattern-based exercises, balance training, aerobic exercise, and cognitive training. Two levels of difficulty (level 1 and level 2) are available, adjustable based on individual ability
Procedure used	Designed to enhance both physical and cognitive health outcomes by promoting user engagement through online videos and interactive elements
Game type	Health promotion game involving multimodal exercise
Game characteristics	
Physical components	Game 1: to enhance physical activity in upper and lower extremities Game 2‐5: to enhance upper extremities and trunk movement
Cognitive components	Game 1: to enhance attention domain Game 2: to enhance working memory, attention, and visuospatial ability Game 3: to enhance executive function, interference inhibition, and attention domains Game 4: to enhance cognitive flexibility and attention domains Game 5: to enhance executive function, working memory, visuospatial ability, and attention domains
Rules and levels	Game 1: follow movement guidelines in online videos. 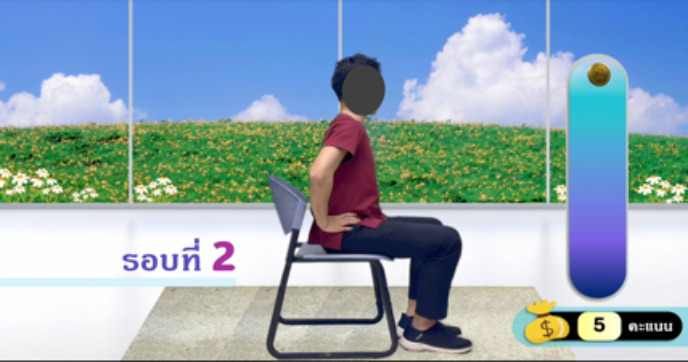 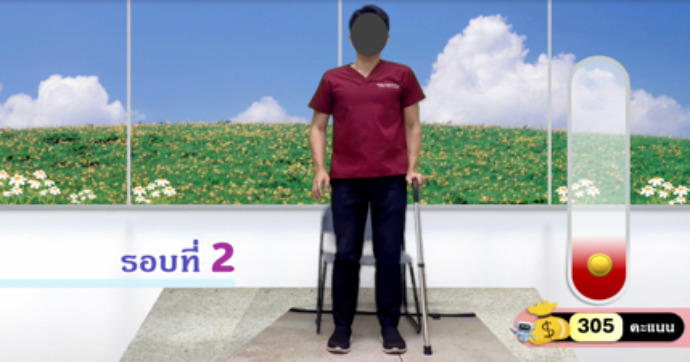 Exercises: seated exercises, sit-to-stand training, PNF pattern-based exercises, balance, and aerobic exercise Level 1: 5 repetitions/set, 2 sets of exercise with combined exercise sets (alternating arm and leg lifts combined with forward arm and leg extensions: 1 min; alternating boxing movements combined with alternating heel-toe tap steps: 40 s). Level 2: 5 repetitions/set, 2 sets of exercise with combined exercise sets (alternating arm and leg lifts combined with forward arm and leg extensions: 2 min; alternating boxing movements combined with alternating heel-toe tap steps: 80 s, preparatory sit-to-stand training and sit-to-stand training for 5 repetitions/set for 3 sets) Game 2: memorize a sequence of numbers displayed on a clock (eg, 5-11-3) and perform corresponding movements by directing their trunk and arm toward each number in the memorized order (eg, move to 5, then 11, and 3) 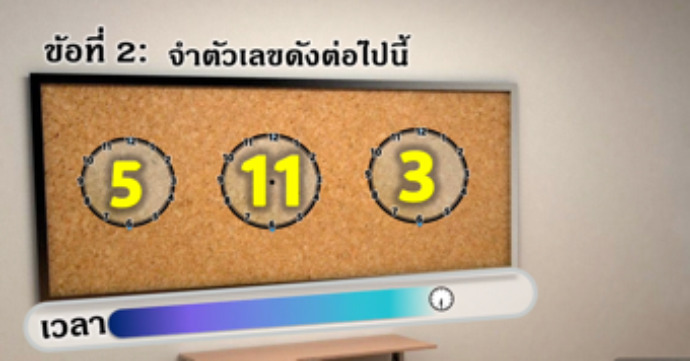 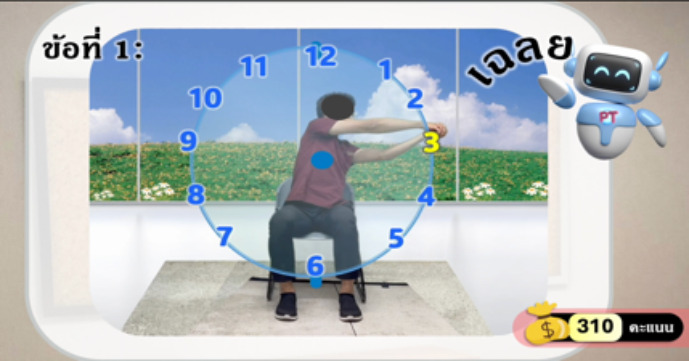 Level 1: 3 Question; Level 2: 4 Question Game 3: memorize fruit and vegetable images within a given time and then collect the corresponding images they remembered, alternating with collecting coins. 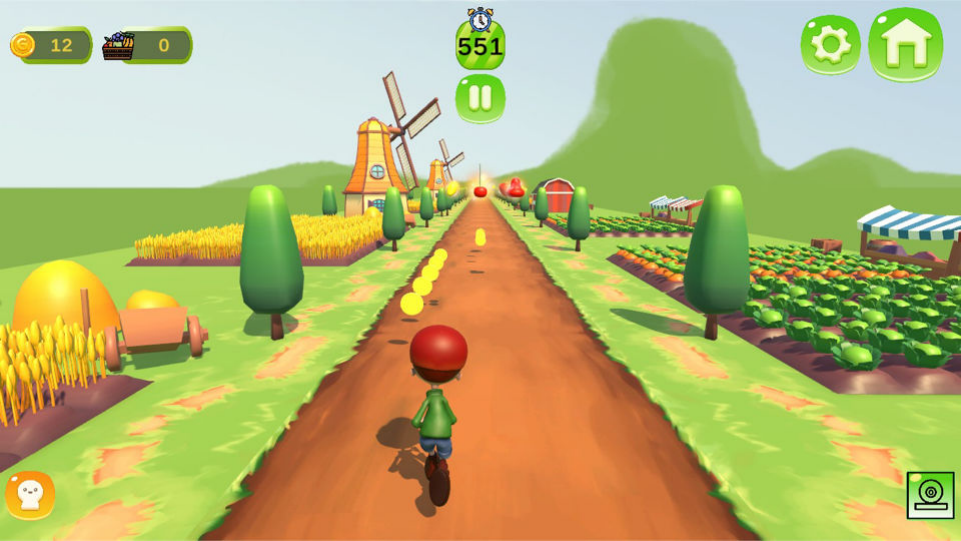 Level 1: Remember 5 pictures in 10 s, 2 nonrelevant pictures Level 2: Remember 5 pictures in 8 s, 5 nonrelevant pictures Game 4: move as quickly as possible to respond to the target based on the image criteria (eg, move toward the target when a mole appears; remain still when a bomb appears) 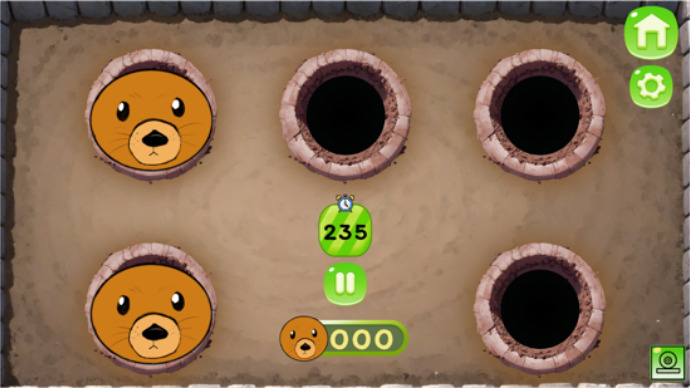 Level 1: Conditions of cognitive stimuli are mole and bombs; the rate of presented cognitive stimuli is 0.2 Level 2: Conditions of cognitive stimuli are mole, mole with hat, and bombs; the rate of presented cognitive stimuli is 0.4 Game 5: remember the characteristics of the ball within a given time, solve mathematical puzzle questions displayed on the screen, and alternate with collecting the balls that matched the ones they had memorized 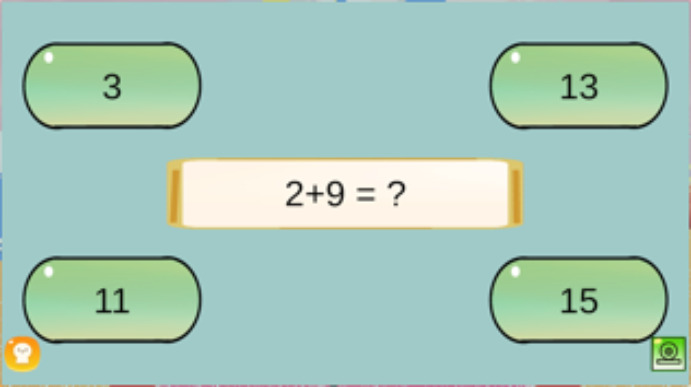 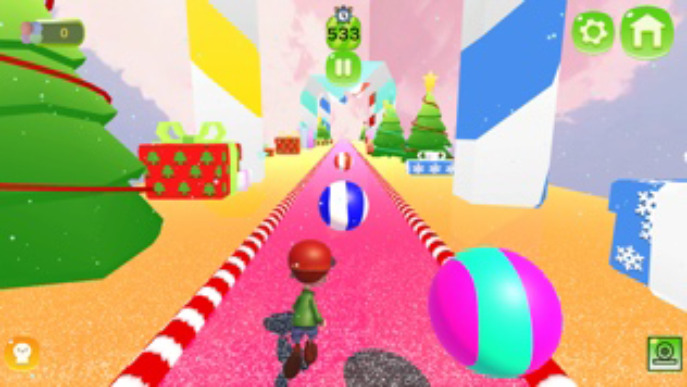 Level 1: 7 Questions (12 s/question), rate of presented stimuli is 0.2 Level 2: 7 Questions (8 s/question), rate of presented stimuli is 0.4
Game mechanics	The program incorporates gamification to support engagement and adherence: Goal setting: clear exercise goals to encourage consistency and motivation Progress bar: visual progress bars help participants monitor their performance Scoring system: real-time performance feedback boosts motivation and supports cognitive engagement Visual and auditory feedback: on-screen cues and sounds provide immediate feedback on performance, reinforcing learning and motivation Interactive elements and cognitive engagement: cognitively engaging tasks and interactive elements are purposefully designed to sustain attention and encourage continuous participation throughout the intervention period
Setting	Indoor space with a plain white background, approximately 3×2 meters in area.
Device	Computer or notebook
Sensor and system	Computer vision-based system
Play time	60 min

a PNF: proprioceptive neuromuscular facilitation.

### Outcome Measures

#### Study Part 1

The usability parameters assessed included enjoyment, RPE, user feedback, satisfaction, and safety. Enjoyment during participation in the multimodal exergame was measured using the Physical Activity Enjoyment Scale (PACES) [37,38]. The PACES comprises 8 items that evaluate enjoyment on a 7-point Likert scale ranging from 1 (strongly disagree) to 7 (strongly agree). Total enjoyment scores were calculated, with higher scores indicating greater enjoyment [37]. For RPE assessment, perceived exertion was measured using the Borg 6‐20 scale [39,40], and ratings of perceived exertion during the exercise session were recorded. Both positive and negative feedback including functionality, software, exercise components, program design, game rules, and difficulty level were recorded and documented to inform program refinement. For satisfaction, participants were instructed to rate their overall satisfaction after using the multimodal exergame on a scale from 1 (very dissatisfied) to 10 (very satisfied). Additionally, safety outcomes were monitored and evaluated through the reporting of adverse events. Any adverse events or incidents occurring during exercise (eg, fatigue, falls, increased muscle tone, and dizziness) or any exercise-related harm were documented and evaluated.

#### Study Part 2

The outcome measures for the feasibility of the home-based multimodal exergame in patients with chronic stroke included physical and cognitive outcomes, enjoyment, adherence, and adverse events. Motor function was assessed using the Short Physical Performance Battery (SPPB) [41]. The battery consists of 3 subtests—standing balance, gait speed, and chair-stand. A total SPPB score was calculated by summing the scores of the subtests, with higher scores indicating better motor function [41]. Functional mobility was measured using the Timed Up and Go test (TUG) [42,43]. Participants were asked to stand up from a chair and move at their maximum but safe pace for 3 meters, then turn and walk back to the seat and sit down. A faster time in the TUG test indicates better functional performance. For cognitive function, global cognition, executive function, and memory were measured using the Montreal Cognitive Assessment (MoCA) test [44], Trail Making Test (TMT A and B) [45], and Digit Span test [46], respectively. Higher scores on the MoCA and Digit Span Test indicate better global cognitive and memory performance, respectively. A smaller difference in completion time between TMT A and B indicates better executive function. Enjoyment was measured using the PACES [37,38]. Additionally, adherence and adverse events were also recorded.

### Data Collection

For study part 1, usability outcomes and adverse events were collected immediately after the exergame session. For study part 2, physical and cognitive outcomes were assessed before and after the 6-week intervention. Program enjoyment was evaluated after 1 week and at the completion of the 6-week training period. Adherence and adverse events were also recorded throughout the study.

### Blinding

Due to the nature of the intervention, participants and researchers administering the intervention were aware of the study procedures. However, outcome assessments were conducted by blinded assessors (CS for cognitive assessment and PE for physical assessment) who were not involved in the intervention delivery.

### Data Diagnostics

All participants completed the study protocol and all outcome assessments, with no missing data or postdata collection exclusions.

### Statistical Analyses

All analyses and statistical calculations were performed using SPSS software (version 25.0, IBM Corporation). The content validity index of the program was determined using the IOC, with an IOC value greater than 0.75 indicating an acceptable level of validity. Descriptive statistics were used to describe the characteristics of participants and usability outcomes. The normality of the data was tested using the Shapiro-Wilk test. Paired sample *t* tests were used to compare the difference in outcome measures between pre- and post–6-week training. Mean differences were calculated, with 95% CIs reported. All data were presented as mean (SD) or n (%). A significant level was set at *P*<.05.

### Ethical Considerations

The study protocol was approved by the Ethics Committee of the Faculty of Associated Medical Sciences, Chiang Mai University (approval AMSEC-67EX-023). All participants provided written informed consent before participation in the study. All collected data were deidentified and handled in accordance with applicable ethical guidelines and data protection regulations to protect participant privacy and confidentiality. Participants received transportation reimbursement of 500 Thai Baht (THB; approximately US $15) in accordance with institutional research regulations. No identifiable personal information or participant images are included in this manuscript.

## Results

### Part 1: Content Validity Index, Features of the Exergame Prototype, and Preliminary Program Usage

#### Overview

The content validity index for each game, based on the average IOC for objectives and level of difficulty, was 1.00 for game 1, 0.90 for game 2, 0.80 for game 3, 0.90 for game 4, and 0.50 for game 5. The overall content validity index was 0.82, indicating an acceptable level of validity. The lower IOC value for game 5 was related to expert feedback indicating that the sequence and arrangement of answer choices on the screen could be confusing and might increase task difficulty beyond the intended objectives of the game. Based on the expert feedback, the design and presentation sequence of game 5 were revised to enhance clarity and usability before inclusion in the final prototype.

The multimodal exergame comprised 5 exergame modes, designed to enhance both physical and cognitive function of individuals with stroke. Each exergame had 2 levels of difficulty, with complexity progressing through an increased number of repetitions and exercise steps, variations in movement direction, extended training duration, increased cognitive load and attentional demands, and reduced resting periods. Additionally, gamification elements such as real-time feedback, scoring systems, performance levels, rewards, and visual and auditory cues were integrated to enhance user motivation, engagement, and adherence to the training regimen. A summary of the consensus features of the home-based multimodal exergame is presented in Table 1.

#### Participant Characteristics

A total of 5 patients with chronic stroke were enrolled in study part 1 to determine the program usage. The mean age of participants was 56.40 (SD 11.37) years, time since stroke onset was 39.40 (SD 33.34) months, the average number of medications was 1.80 (SD 1.64), and BMI was 24.85 (SD 4.36) kg/m². All participants were male, with 2 diagnosed with hemorrhagic stroke and 3 with ischemic stroke. Most participants (4/5, 80%) had left-side paresis. None of the participants had previous experience using exergames. The demographic characteristics of participants are presented in Table 2.

**Table 2.** Demographic characteristics of participants in study phase 1 (n=5).

Characteristics	Statistical value
Age (y), mean (SD)	56.40 (11.37)
Sex, n (%)	
Male	5 (100)
Female	0 (0)
Weight (kg), mean (SD)	68.55 (14.66)
Height (cm), mean (SD)	165.80 (6.14)
BMI (kg/cm^2^), mean (SD)	24.85 (4.36)
Time post onset (months), mean (SD)	39.40 (33.34)
Types, n (%)	
Hemorrhagic	2 (40)
Ischemic	3 (60)
Paretic side, n (%)	
Left	4 (80)
Right	1 (20)
Types of medication (types), mean (SD)	1.80 (1.64)
MSET10^a^, mean (SD)	26.40 (1.50)
Education status, n (%)	
High school graduate	2 (40)
University graduate	2 (40)
Postgraduate	1 (20)

a MSET10: Mental State Examination T10

#### Preliminary Usage Outcomes

The average total enjoyment score after exercise as measured by PACES was 47.80 (SD 7.50). The average score for each item was 5.97 (SD 0.94), with subitem averages ranging from 5.60 to 6.40 (Table 3). For the ratings of perceived exertion, 3 participants reported a score of 13, and 2 participants reported a score of 14 (Table 4). The average perceived exertion rating was 13.40 (SD 0.55; Table 4). Regarding satisfaction, the average score was 8.60 (SD 1.34), with scores ranging from 7 to 10 (Table 4). Overall feedback indicated that most participants found the functionality of the multimodal exergame user-friendly and easy to understand. Most participants also reported that the exergame software was generally well-designed to provide an optimal level of difficulty (neither too difficult nor too simple), and that the game featured clear illustrations and vibrant cartoon graphics. All participants reported that all game levels are well-designed and provide an appropriate challenge. As for the negative feedback, two participants reported that the sensor occasionally responded slowly and that the fruit images in the game were difficult to see clearly. Additionally, 1 participant noted that some exercise movements (eg, alternating between toe taps and heel taps) were too complicated, while another mentioned that the game rule instructions were confusing when playing for the first time. User feedback and suggestions, including both positive and negative aspects, are summarized in Table 5. Importantly, no musculoskeletal complaints, injuries, falls, or adverse events were reported during the multimodal exergame training.

**Table 3.** PACES^a^ rating scores (n=5).

PACES items	Response rating					
	ID01	ID02	ID03	ID04	ID05	Mean (SD)
I ﬁnd it pleasurable	7	6	7	6	6	6.40 (0.55)
It’s a lot of fun	5	5	7	5	6	5.60 (0.89)
It’s very pleasant	6	6	7	6	6	6.20 (0.45)
It’s very invigorating	6	6	7	5	6	6.00 (0.71)
It’s very gratifying	7	5	7	5	5	5.80 (1.10)
It’s very exhilarating	7	6	7	5	6	6.20 (0.84)
It’s very stimulating	7	6	7	2	6	5.60 (2.07)
It’s very refreshing	7	7	7	2	7	6.00 (2.24)
Rating scale of all items (total points: 56)	52	47	56	36	48	For each item: 5.97 (0.94) For all items: 47.80 (7.50)

a PACES: Physical Activity Enjoyment Scale.

**Table 4.** Satisfaction scores and ratings of perceived exertion (n=5).

ID	Ratings of perceived exertion^a^ (ranged from 6 to 20)	Overall satisfaction score^b^ (maximum score: 10)
ID01	13.00	10.00
ID02	14.00	7.00
ID03	13.00	10.00
ID04	13.00	8.00
ID05	14.00	8.00

a mean 13.40, SD 0.55.

b mean 8.60, SD 1.34.

**Table 5.** The feedback and suggestions from all participants (n=5).

Criteria	Positive feedback	Negative feedback
Functionality	User-friendly and easy to understand (n=3)	The sensor occasionally responds slowly (n=2)
Software	The program is generally designed to provide an optimal level of difficulty (neither too difficult nor too simple) (n=3)	—^a^
Exercise and cognitive program	The exercise movements are generally easy to follow (n=2)	Some movements are too complicated, such as alternating between toe taps and heel taps (n=1)
Design	The exercise game has clear illustrations and is easy to follow (n=2); it also features vibrant and engaging cartoon graphics (n=1)	Some images, such as the fruits in the game, are too small and difficult to see clearly (n=2)
Game rule	The rules and instructions are clearly explained and easy to understand (n=3)	Some instructions are initially confusing but become clear after subsequent attempts (n=1)
Level	All game levels are well-designed and provide an appropriate challenge (n=5)	—

a Not available.

### Part 2: Feasibility of the Home-Based Multimodal Exergame

#### Participant Characteristics

A total of 9 patients with chronic stroke were enrolled in study part 2. The mean age of participants was 53.67 (SD 11.70, range 39-68) years, time post stroke onset was 75.00 (SD 101.99, range 6-264) months, number of medications was 1.56 (SD 1.51, range 0-4), and the level of education was 13.33 (SD 4.33, range 6-21) years. Of the 9 participants, 7 were male and 2 were female; 4 were diagnosed with hemorrhagic stroke, while 5 were diagnosed with ischemic stroke. Moreover, 5 participants presented with left-side paresis, and 4 with right-side paresis. The demographic characteristics of the participants are summarized in Table 6.

**Table 6.** Demographic characteristics of participants in study phase 2 (n=9).

Characteristics	Statistical value
Age (y), mean (SD)	53.67 (11.70)
Sex, n (%)	
Male	7 (77.78)
Female	2 (22.22)
Weight (kg), mean (SD)	67.54 (13.31)
Height (cm), mean (SD)	165.00 (10.79)
BMI (kg/cm^2^), mean (SD)	24.75 (3.79)
Time post onset (months), mean (SD)	75.00 (101.99)
Types, n (%)	
Hemorrhagic	4 (44.44)
Ischemic	5 (55.56)
Paretic side, n (%)	
Left	5 (55.56)
Right	4 (44.44)
Types of medication (types), mean (SD)	1.56 (1.51)
MSET10^a^, mean (SD)	25.67 (1.87)
Years of education, mean (SD)	13.33 (4.33)

a MSET10: Mental State Examination T10.

#### Feasibility Outcomes, Adherence, and Adverse Events

Results indicated that improvements in the MoCA score (mean difference=−3.44, 95% CI −5.66 to−1.23; *P*=.007), TMT B completion time (mean difference=38.75, 95% CI 8.27-69.22; *P*=.02), and TMT B-A (mean difference=38.43, 95% CI 3.78-73.08; *P*=.03) were observed from pretest to posttest (Table 7). No significant changes were observed in the SPPB score, time in TUG, time to complete the TMT A, digit span sum score, and PACES sum score posttest compared with pretest (mean difference=−0.22, 95% CI −1.22 to 0.78; *P*=.62; mean difference=2.31, 95% CI −1.53 to 6.15; *P*=.20; mean difference=0.32, 95% CI −14.14 to 14.77; *P*=.96; mean difference=−0.22, 95% CI −1.80 to 1.35; *P*=.75; and mean difference=−1.78, 95% CI −6.99 to 3.43; *P*=.45, respectively; Table 7). Additionally, the average exercise adherence rate was 85.19%. Throughout the 6-week training period, no adverse events, injuries, or falls were reported.

**Table 7.** Results of feasibility outcomes at pretest and posttest (n=9).

Feasibility outcomes	Pretest, mean (SD)	Posttest, mean (SD)	*P* value	Mean differences^a^ (95% CI)
SPPB^b^ score	6.56 (1.67)	6.78 (2.11)	.62	−0.22 (−1.22 to 0.78)
TUG^c^ (sec)	22.82 (12.71)	20.51 (9.30)	.20	2.31 (−1.53 to 6.15)
MoCA^d^ score	20.00 (3.84)	23.44 (2.83)	.007^e^	−3.44 (−5.66 to −1.23)
TMT A^f^ (sec)	67.26 (15.93)	66.94 (18.11)	.96	0.32 (−14.14 to 14.77)
TMT B^g^ (sec)	188.73 (76.58)	149.99 (47.14)	.02^e^	38.75 (8.27 to 69.22)
TMT B-A^h^ (sec)	121.48 (76.97)	83.05 (40.31)	.03^e^	38.43 (3.78 to 73.08)
Digit span (sum score)	11.89 (3.48)	12.11 (2.62)	.75	−0.22 (−1.80 to 1.35)
PACES^i^ (sum score)	49.22 (6.76)	51.00 (5.59)	.45	−1.78 (−6.99 to 3.43)

a Mean differences were calculated as pretest minus posttest values.

b SPPB: Short Physical Performance Battery.

c TUG: Timed Up and Go test.

d MoCA: The Montreal Cognitive Assessment test.

e *P<.05 *(Paired sample *t*-tests).

f TMT A: Trail Making Test A.

g TMT B: Trail Making Test B.

h TMT B-A: Trail Making Test B-A.

i PACES: Physical Activity Enjoyment Scale.

## Discussion

### Principal Findings

To the best of our knowledge, this intervention is the first prototype of an interactive, multimodal exercise program incorporating gamification and augmented feedback, specifically designed to target both cognitive and physical outcomes in individuals with chronic stroke residing at home or in remote areas. Findings from this study demonstrated that the preliminary use of the developed exergame prototype showed high content validity (IOC=0.82) and was enjoyable, easily accessible, user-friendly, and safe for patients with chronic stroke. Feasibility outcomes further support the implementation of this program, as reflected by a high adherence rate, absence of adverse events, and sustained enjoyment throughout the training period. Collectively, these findings suggest that the multimodal exergame is both safe and feasible for use in individuals with chronic stroke and may offer potential benefits for cognitive outcomes following 6 weeks of training.

Stroke rehabilitation interventions and poststroke exercise programs frequently encounter challenges, such as long travel distances, a lack of available health care providers, high dropout rates, and low motivation [13,14], all of which contribute to physical inactivity [11]. To mitigate rehabilitation barriers, this study developed and implemented a gamified physical-cognitive exercise program delivered through practical and accessible formats, including online videos and home-based training. Based on a synthesis of existing evidence [33,34,47,48] and a development brainstorming process, both physical and cognitive interventions for stroke rehabilitation were developed. All essential exercises for stroke rehabilitation were incorporated into the multimodal exercise program. Findings demonstrated that the content validity index of the overall program was high, indicating that the content of the home-based multimodal exergame is appropriate for individuals with chronic stroke. Additionally, interactive game-based training has emerged as a promising strategy to enhance user engagement, potentially leading to improved adherence and more favorable intervention outcomes [15,21,22]. The program in this study was designed according to gamification principles to promote user engagement and enjoyment. This study revealed that all participants reported high satisfaction and enjoyment with the program. The average score on PACES for each item was 5.97 ranged from 5.60 to 6.40, reflecting positive emotional responses and enjoyment during the combined physical and cognitive activities. The use of gamification elements may enhance enjoyment and promote long-term participation by aligning physical and cognitive challenges with game-like experiences [34,49,50]. These findings align with previous research demonstrating that gamification elements, such as goals, rules, and rewards, improved intrinsic motivation, satisfaction, and enjoyment in exercise programs [34,49-51]. Furthermore, the use of augmented feedback may provide extrinsic information during training sessions and plays a crucial role in enhancing motivation in individuals with stroke [18].

Usability feedback from 5 patients with chronic stroke indicated the multimodal exergame was easy to use, appropriately challenging, and included exercise movements that were suitable and easy to follow. The results were in line with the previous study [34,52] and suggested that the positive feedback from users unfamiliar with new technologies may be due to the user-friendly interface and appropriately challenging task levels incorporated into the intervention design. However, some users reported occasional delays in game graphics and sensor detection; exercise movements, such as alternating between toe taps and heel taps, were too complicated, and some instructions were unclear. In response to negative feedback from participants, technical adjustments were implemented to enhance sensor performance and reduce system latency. Additionally, minor revisions to graphics and certain instructions were made based on consensus from the expert panel and research team. The finalized version of the intervention program was developed through a rigorous process. Consequently, the preliminary prototype of the multimodal exergame demonstrated satisfactory scientific validity, aligning with constructs relevant to specific clinical and research objectives in poststroke rehabilitation.

The American Stroke Association demonstrated that encouraging physical activity among survivors of stroke should focus on moderate-intensity of exercises and managing risk factors to prevent secondary stroke and improve cardiovascular health [6]. To confirm exercise intensity, this study used the Borg scale for subjective assessment [53]. It has been documented that the RPE is a simple and practical method that does not require special equipment to measure an individual’s subjective level of exertion and is used to ensure that exercise intensity remains within tolerable limits [6,53]. The results of this study showed that the average perceived exertion rating during exercise was 13.40, indicating moderate-intensity exercise. Previous studies showed that exercise at moderate intensity promoted health benefits in patients with stroke [54,55]. Thus, the prototype of multicomponent exercise at moderate intensity could provide opportunities to support stroke recovery. Additionally, integrating objective measures (eg, heart rate monitoring via wearable devices) would further enhance the precision of intensity monitoring and enable more personalized and adaptive progression of the exercise program. Furthermore, combining perceived exertion with heart rate–based targets may facilitate automated adjustment of exercise intensity across sessions.

Emerging evidence suggests that multimodal interventions integrating physical and cognitive components may confer broader benefits than single-domain training in older adults [56,57]. In addition, research evidence has demonstrated that multimodal exercise improved health outcomes in older adult patients with stroke and frailty [7] and older adults with dementia [8]. In this feasibility study, participants demonstrated improvements in global cognition and executive function following a 6-week home-based multimodal exergame. However, these findings should be interpreted with caution due to the small sample size and the absence of a control group, which limit causal inference and increase the risk of overestimating intervention effects.

The observed trends in cognitive outcomes may be partly explained by the design of the intervention. Specifically, the exergame tasks were structured to engage multiple domains of cognition, with a particular emphasis on executive processes such as attention, inhibition, and cognitive flexibility. In contrast, memory-related tasks were less frequently represented and primarily limited to working memory components. In addition, the progression from lower to higher difficulty levels incorporated increased cognitive load, shorter response times, and greater exposure to distracting stimuli. These features likely required maintained attention and adaptive control, which may preferentially stimulate executive function [58,59]. Nevertheless, given the exploratory nature of this study, these mechanistic interpretations remain speculative and should be confirmed in controlled trials.

In contrast to the cognitive findings, motor performance, as assessed by the SPPB and TUG, did not show significant changes following the intervention. This lack of improvement may be related to several characteristics of the training program. To prioritize the safety of home-based and remote training, the intervention primarily emphasized seated activities and sit-to-stand exercises, with limited incorporation of dynamic balance, gait training, or higher-intensity lower extremity tasks. As a result, the stimulus may not have been sufficiently specific or intense to elicit measurable improvements in functional mobility outcomes that require integrated components of balance, walking speed, and transitional movements. Furthermore, the outcome measures themselves may have influenced the findings. Both SPPB and TUG capture complex, whole-body functional performance, including gait and dynamic balance [41-43], which were not extensively targeted in the intervention. Therefore, the transfer of training effects to these measures may have been limited. It is also possible that the relatively short intervention duration and limited sample size reduced the statistical power to detect changes in both motor performance and memory outcomes. Importantly, no adverse events were reported during training with the home-based multimodal exergame. After evaluating the feasibility of the program, the total PACES score was maintained over the training period, and a high adherence rate was presented. This finding highlights the safety and feasibility of remote, technology-assisted interventions for individuals with chronic stroke. Additionally, participants were able to engage consistently with the exergame protocol in remote areas and their home environments.

Taken together, while the findings support the feasibility of a home-based multimodal exergame intervention and suggest potential cognitive benefits, they should be interpreted as preliminary. Future studies using randomized controlled designs, larger sample sizes, and longer intervention periods are needed to establish efficacy. Additionally, incorporating more task-specific and higher-intensity motor components, as well as selecting outcome measures that are closely aligned with the training stimuli, may help clarify the extent to which such interventions can improve both cognitive and motor function in individuals with chronic stroke.

### Limitations

This study has several limitations that should be acknowledged. All participants demonstrated normal range of cognitive function, as determined by a screening tool. In addition, most participants had an educational background of high-school level or higher. Therefore, the findings primarily reflect the experiences of this specific group and may not capture the perspectives of individuals with different cognitive profiles or those with lower educational attainment. Although RPE is well-documented as a practical method for measuring an individual’s subjective level of exertion during exercise, incorporating objective measurements (eg, polar heart rate monitors) would provide a more precise and comprehensive assessment of exercise intensity, while enabling personalized intensity adjustment in future studies. Furthermore, participants’ technology literacy was not formally assessed and may have influenced the study outcomes, particularly usability, satisfaction, and adherence. As this study primarily assessed the content validity, usability, and feasibility of the intervention program in patients with chronic stroke, the effectiveness of the system and program requires further investigation through large-scale, methodologically rigorous clinical trials to establish definitive clinical outcomes.

### Conclusions

The home-based multimodal exergame demonstrated acceptable content validity, usability, and feasibility in individuals with stroke. Unlike conventional home exercise programs, this multimodal exergame integrates both physical and cognitive training into an interactive home-based and remote rehabilitation platform for patients with chronic stroke. The intervention was found to be enjoyable, accessible, user-friendly, and safe, while showing potential benefits for cognitive outcomes, maintaining exercise enjoyment, and promoting exercise adherence throughout the training period. This preliminary evaluation suggests that the home-based multimodal exergame is a promising rehabilitation tool that may enhance patient engagement, support long-term rehabilitation participation, and help address barriers to stroke rehabilitation, particularly for individuals living in underserved or remote areas.

## Abbreviations

COMBO+SET: combined exercise set
IOC: item-objective congruence
MoCA: Montreal Cognitive Assessment
PACES: Physical Activity Enjoyment Scale
PNF: proprioceptive neuromuscular facilitation
RPE: rating of perceived exertion
SPPB: Short Physical Performance Battery
TMT: Trail Making Test
TUG: Timed Up and Go test

## References

[1] Sacco RL, Kasner SE, Broderick JP (2013). An updated definition of stroke for the 21st century. Stroke.

[2] Feigin VL, Stark BA, Johnson CO (2021). Global, regional, and national burden of stroke and its risk factors, 1990–2019: a systematic analysis for the Global Burden of Disease Study 2019. Lancet Neurol.

[3] Einstad MS, Saltvedt I, Lydersen S (2021). Associations between post-stroke motor and cognitive function: a cross-sectional study. BMC Geriatr.

[4] Lin H, Liu H, Dai Y (2022). Effect of physical activity on cognitive impairment in patients with cerebrovascular diseases: a systematic review and meta-analysis. Front Neurol.

[5] Meilani E, Yojana E, Prayitno DA, Ahmad MA (2023). The effect of multimodal therapy on balance outcomes of stroke survivors: a systematic review. MPJ.

[6] Billinger SA, Arena R, Bernhardt J (2014). Physical activity and exercise recommendations for stroke survivors: a statement for healthcare professionals from the American Heart Association/American Stroke Association. Stroke.

[7] Luo Y, Hao J, Zhu L (2024). Effects of multicomponent exercise nursing intervention in elderly stroke patients with frailty: a randomized controlled trial. Front Med (Lausanne).

[8] Borges-Machado F, Silva N, Farinatti P, Poton R, Ribeiro Ó, Carvalho J (2021). Effectiveness of multicomponent exercise interventions in older adults with dementia: a meta-analysis. Gerontologist.

[9] Pellicer MG, Lusar AC, Casanovas JM, Ferrer BCS (2017). Effectiveness of a multimodal exercise rehabilitation program on walking capacity and functionality after a stroke. J Exerc Rehabil.

[10] English C, Healy GN, Coates A, Lewis LK, Olds T, Bernhardt J (2016). Sitting time and physical activity after stroke: physical ability is only part of the story. Top Stroke Rehabil.

[11] Botö S, Buvarp DJ, Hansson PO, Sunnerhagen KS, Persson CU (2021). Physical inactivity after stroke: Incidence and early predictors based on 190 individuals in a 1-year follow-up of the Fall Study of Gothenburg. J Rehabil Med.

[12] Yao M, Chen J, Jing J, Sheng H, Tan X, Jin J (2017). Defining the rehabilitation adherence curve and adherence phases of stroke patients: an observational study. Patient Prefer Adherence.

[13] Nicholson S, Sniehotta FF, van Wijck F (2013). A systematic review of perceived barriers and motivators to physical activity after stroke. Int J Stroke.

[14] Demiris G, Shigaki CL, Schopp LH (2005). An evaluation framework for a rural home-based telerehabilitation network. J Med Syst.

[15] Chan KGF, Jiang Y, Choo WT, Ramachandran HJ, Lin Y, Wang W (2022). Effects of exergaming on functional outcomes in people with chronic stroke: a systematic review and meta-analysis. J Adv Nurs.

[16] Jia C, Liu X, Ning L, Ge L (2025). The effects of augmented reality on rehabilitation of stroke patients: a systematic review and meta-analysis with trial sequential analysis. J Clin Nurs.

[17] Johansson GM, Öhberg F (2025). Augmented feedback in post-stroke gait rehabilitation derived from sensor-based gait reports-a longitudinal case series. Sensors (Basel).

[18] Hornby TG, Reisman DS, Ward IG (2020). Clinical practice guideline to improve locomotor function following chronic stroke, incomplete spinal cord injury, and brain injury. J Neurol Phys Ther.

[19] Alhirsan SM, Capó-Lugo CE, Hurt CP, Uswatte G, Qu H, Brown DA (2023). The immediate effects of different types of augmented feedback on fast walking speed performance and intrinsic motivation after stroke. Arch Rehabil Res Clin Transl.

[20] Khan A, Imam YZ, Muneer M, Al Jerdi S, Gill SK (2024). Virtual reality in stroke recovery: a meta-review of systematic reviews. Bioelectron Med.

[21] Stanmore E, Stubbs B, Vancampfort D, de Bruin ED, Firth J (2017). The effect of active video games on cognitive functioning in clinical and non-clinical populations: a meta-analysis of randomized controlled trials. Neurosci Biobehav Rev.

[22] Wu S, Jo EA, Ji H (2019). Exergaming improves executive functions in patients with metabolic syndrome: randomized controlled trial. JMIR Serious Games.

[23] Kamnardsiri T, Kumfu S, Munkhetvit P, Boripuntakul S, Sungkarat S (2024). Home-based, low-intensity, gamification-based, interactive physical-cognitive training for older adults using the ADDIE model: design, development, and evaluation of user experience. JMIR Serious Games.

[24] Hung JW, Chou CX, Hsieh YW (2014). Randomized comparison trial of balance training by using exergaming and conventional weight-shift therapy in patients with chronic stroke. Arch Phys Med Rehabil.

[25] Chen SC, Lin CH, Su SW, Chang YT, Lai CH (2021). Feasibility and effect of interactive telerehabilitation on balance in individuals with chronic stroke: a pilot study. J Neuroeng Rehabil.

[26] Gauthier LV, Nichols-Larsen DS, Uswatte G (2022). Video game rehabilitation for outpatient stroke (VIGoROUS): a multi-site randomized controlled trial of in-home, self-managed, upper-extremity therapy. EClinicalMedicine.

[27] Liu-Ambrose T, Falck RS, Dao E (2022). Effect of exercise training or complex mental and social activities on cognitive function in adults with chronic stroke: a randomized clinical trial. JAMA Netw Open.

[28] Song YY, Sun WJ, Wang C, Tian YM, Liu H, Jiang Y (2023). Effects of multicomponent exercise on quality of life, depression and anxiety among stroke survivors: a systematic review and meta-analysis. J Clin Nurs.

[29] Wongpakaran N (2021). Mental State Examination T10 (MSET10).

[30] Boongerd P (2018). Interesting topic about dementia. Dementia Association of Thailand.

[31] Nucita A, Iannizzotto G, Perina M, Romano A, Fabio RA (2023). Telerehabilitation with computer vision-assisted markerless measures: a pilot study with Rett syndrome patients. Electronics (Basel).

[32] He S, Meng D, Wei M, Guo H, Yang G, Wang Z (2024). Proposal and validation of a new approach in tele-rehabilitation with 3D human posture estimation: a randomized controlled trial in older individuals with sarcopenia. BMC Geriatr.

[33] Keawtep P, Kamnardsiri T, Boripuntakul S, Wichayanrat W, Worakul P, Sungkarat S (2023). Feasibility of internet-based physical-cognitive exercise for health benefits of middle-aged obese women. J Prim Care Community Health.

[34] Kamnardsiri T, Phirom K, Boripuntakul S, Sungkarat S (2021). An interactive physical-cognitive game-based training system using Kinect for older adults: development and usability study. JMIR Serious Games.

[35] Harte R, Glynn L, Rodríguez-Molinero A (2017). A human-centered design methodology to enhance the usability, human factors, and user experience of connected health systems: a three-phase methodology. JMIR Hum Factors.

[36] Turner RC, Carlson L (2003). Indexes of item-objective congruence for multidimensional items. International Journal of Testing.

[37] Raedeke TD (2007). The relationship between enjoyment and affective responses to exercise. J Appl Sport Psychol.

[38] Kendzierski D, DeCarlo KJ (1991). Physical Activity Enjoyment Scale: two validation studies. J Sport Exerc Psychol.

[39] Borg GAV (1982). Psychophysical bases of perceived exertion. Medicine & Science in Sports & Exercise.

[40] Kossi O, Lacroix J, Compagnat M, Daviet JC, Mandigout S (2021). Perceived exertion and energy expenditure during physical activities in healthy young people and older adults. Folia Med (Plovdiv).

[41] Stookey AD, Katzel LI, Steinbrenner G, Shaughnessy M, Ivey FM (2014). The Short Physical Performance Battery as a predictor of functional capacity after stroke. J Stroke Cerebrovasc Dis.

[42] Shumway-Cook A, Brauer S, Woollacott M (2000). Predicting the probability for falls in community-dwelling older adults using the Timed Up & Go test. Phys Ther.

[43] Persson CU, Danielsson A, Sunnerhagen KS, Grimby-Ekman A, Hansson PO (2014). Timed Up & Go as a measure for longitudinal change in mobility after stroke - Postural Stroke Study in Gothenburg (POSTGOT). J Neuroeng Rehabil.

[44] Nasreddine ZS, Phillips NA, Bédirian V (2005). The Montreal Cognitive Assessment, MoCA: a brief screening tool for mild cognitive impairment. J Am Geriatr Soc.

[45] Tombaugh TN (2004). Trail Making Test A and B: normative data stratified by age and education. Arch Clin Neuropsychol.

[46] Fink A, Hemmy S, MacDonald R (2014). Cognitive outcomes after cardiovascular procedures in older adults: a systematic review [Internet].

[47] Salgueiro C, Urrútia G, Cabanas-Valdés R (2022). Influence of core-stability exercises guided by a telerehabilitation app on trunk performance, balance and gait performance in chronic stroke survivors: a preliminary randomized controlled trial. Int J Environ Res Public Health.

[48] Park SJ, Oh S (2020). Effect of diagonal pattern training on trunk function, balance, and gait in stroke patients. Applied Sciences.

[49] Moholdt T, Weie S, Chorianopoulos K, Wang AI, Hagen K (2017). Exergaming can be an innovative way of enjoyable high-intensity interval training. BMJ Open Sport Exerc Med.

[50] Fanaroff AC, Patel MS, Chokshi N (2024). Effect of gamification, financial incentives, or both to increase physical activity among patients at high risk of cardiovascular events: the BE ACTIVE randomized controlled trial. Circulation.

[51] Krause L, Farrow D, Pinder R, Buszard T, Kovalchik S, Reid M (2019). Enhancing skill transfer in tennis using representative learning design. J Sports Sci.

[52] Lu MH, Lin W, Yueh HP (2017). Development and evaluation of a cognitive training game for older people: a design-based approach. Front Psychol.

[53] Billinger SA, Boyne P, Coughenour E, Dunning K, Mattlage A (2015). Does aerobic exercise and the FITT principle fit into stroke recovery?. Curr Neurol Neurosci Rep.

[54] Hill G, Johnson F, Uy J (2023). Moderate intensity aerobic exercise may enhance neuroplasticity of the contralesional hemisphere after stroke: a randomised controlled study. Sci Rep.

[55] Constans A, Pin-Barre C, Temprado JJ, Decherchi P, Laurin J (2016). Influence of aerobic training and combinations of interventions on cognition and neuroplasticity after stroke. Front Aging Neurosci.

[56] Cadore EL, Rodríguez-Mañas L, Sinclair A, Izquierdo M (2013). Effects of different exercise interventions on risk of falls, gait ability, and balance in physically frail older adults: a systematic review. Rejuvenation Res.

[57] Sáez de Asteasu ML, Martínez-Velilla N, Zambom-Ferraresi F, Casas-Herrero Á, Izquierdo M (2017). Role of physical exercise on cognitive function in healthy older adults: a systematic review of randomized clinical trials. Ageing Res Rev.

[58] Schättin A, Arner R, Gennaro F, de Bruin ED (2016). Adaptations of prefrontal brain activity, executive functions, and gait in healthy elderly following exergame and balance training: a randomized-controlled study. Front Aging Neurosci.

[59] Kumfu S, Kamnardsiri T, Boripuntakul S, Sungkarat S (2025). Feasibility of home-based, low-intensity exergame on cognitive function of older adults with mild cognitive impairment. Digit Health.

